# Nutritional Risk Index as a Prognostic Factor Predicts the Clinical Outcomes in Patients With Stage III Gastric Cancer

**DOI:** 10.3389/fonc.2022.880419

**Published:** 2022-05-13

**Authors:** Haibin Song, Hongkai Sun, Laishou Yang, Hongyu Gao, Yongkang Cui, Chengping Yu, Haozhi Xu, Linqiang Li

**Affiliations:** ^1^ Department of Gastrointestinal Surgery, Harbin Medical University Cancer Hospital, Harbin Medical University, Harbin, China; ^2^ Department of Anesthesiology, Hulunbeier People’s Hospital, Hulunbeier, China; ^3^ Department of General Surgery, The First Affiliated Hospital of Harbin Medical University, Harbin Medical University, Harbin, China; ^4^ Key Laboratory of Hepatosplenic Surgery, Ministry of Education, Harbin Medical University, Harbin, China

**Keywords:** gastric cancer, nutritional risk index, nutrition, inflammation, prognosis

## Abstract

**Objective:**

This study is aimed to determine the potential prognostic significance of nutritional risk index (NRI) in patients with stage III gastric cancer.

**Methods:**

A total of 202 patients with stage III gastric cancer were enrolled in this study. NRI was an index based on ideal body weight, present body weight, and serum albumin levels. All patients were divided into two groups by receiver operating characteristic curve: low NRI group (NRI<99) and high NRI group (NRI≥99). The relationship between NRI and clinicopathologic characteristics was evaluated by Chi-square test. The clinical survival outcome was analyzed by Kaplan-Meier method and compared using log-rank test. The univariate and multivariate analyses were used to detect the potential prognostic factors. A nomogram for individualized assessment of disease-free survival (DFS) and overall survival (OS). The calibration curve was used to evaluate the performance of the nomogram for predicted and the actual probability of survival time. The decision curve analysis was performed to assess the clinical utility of the nomogram by quantifying the net benefits at different threshold probabilities.

**Results:**

The results indicated that NRI had prognostic significance by optimal cutoff value of 99. With regard to clinicopathologic characteristics, NRI showed significant relationship with age, weight, body mass index, total protein, albumin, albumin/globulin, prealbumin, glucose, white blood cell, neutrophils, lymphocyte, hemoglobin, red blood cell, hematocrit, total lymph nodes, and human epidermal growth factor receptor 2 (P<0.05). Through the univariate and multivariate analyses, NRI, total lymph nodes, and tumor size were identified as the independent factor to predict the DFS and OS. The nomogram was used to predict the 1-, 3-, and 5-year survival probabilities, and the calibration curve showed that the prediction line matched the reference line well for 1-, 3-, and 5-year DFS and OS. Furthermore, the decision curve analysis also showed that the nomogram model yielded the best net benefit across the range of threshold probability for 1-, 3-, 5-year DFS and OS.

**Conclusions:**

NRI is described as the potential prognostic factor for patients with stage III gastric cancer and is used to predict the survival and prognosis.

## Introduction

Gastric cancer is a deadly disease with poor prognosis and remains an unsolved major clinical problem with more than one million new cases throughout the world (1). Gastric cancer is the sixth leading cause of cancer-related morbidity and the third leading cause of cancer-related death worldwide, and the majority of newly diagnosed gastric cancer occurs mainly in Eastern Asia (2). Although early detection and recent improvements in surgery and chemotherapy have improved the clinical outcome, the mortality is still high in patients with advanced gastric cancer and recurrent disease (3). Most cases are diagnosed in the late stage of the disease, resulting in overall poor outcomes, including high intratumor heterogeneity, metastases, and chemotherapeutic resistance (4). In addition to the difference in disease status, nutritional status also plays an important role in influencing the patients’ prognosis, treatment effect, and clinical outcome.

Previous studies have indicated that malnutrition might lead to a poor response to anti-tumor treatment, increase the incidence of postoperative complications, and result in an unsatisfactory survival prognosis (5). As a result of the imbalance between intake of nutrients and requirements, malnutrition is a common risk factor for postoperative complications and poor prognosis in patients with gastric cancer (6). Cachexia is a complex multifactorial syndrome that affects 50% - 80% of cancer patients and is also associated with 20% - 40% of cancer deaths (7). Early assessment and management of nutrition for gastric cancer patients can improve clinical outcomes. Currently known indicators reflecting the nutritional status of patients include Nutritional Risk Screening (NRS), malnutrition screening tool (MST), Naples Prognostic Score (NPS), prognostic nutritional index (PNI), patient-generated subjective global assessment (PG-SGA), and body mass index (BMI) (8–13). These indexes are the common screening tools, and each one possesses some benefits when screening patients for malnutrition. Recently, an increasing number of studies report that the nutritional risk index (NRI), which is established based on the patients’ ideal body weight, present body weight, and serum albumin levels, represents a new nutrition-related prognostic scoring system (14). Researchers have shown that NRI had prognostic value for breast cancer, esophageal cancer, and oral cancer (15–17). This emerged indicator takes into account the effects of nutritional status and systemic inflammation condition on cancer prognosis. Hence, NRI is superior to other single nutritional or inflammatory markers. Several studies have also indicated that NRI was related to gastric cancer. In Oh CA and colleagues’ study, they found that NRI was a predictor in postoperative wound complications after gastrectomy and played an important role in the development of wound complications with malnutrition immediately after surgery (18). Another study has shown that Geriatric Nutritional Risk Index (GNRI) was useful in predicting postoperative complications of elderly patients with GC undergoing gastrectomy, and emerged as an independent predictor of postoperative complications (19). Another study investigated whether the GNRI was affected by the number of remaining teeth, occlusal support status, and denture use in gastric cancer patients, and the result showed that GNRI was associated with the occlusal support level but not with denture use (20). However, this indicator remains limited for patients with stage III gastric cancer. As a result, the present retrospective cohort study aims to determine the prognostic significance of NRI in patients with stage III gastric cancer and to investigate the correlation between NRI and clinicopathological characteristics.

## Materials and Methods

### Study Population

The retrospective study included patients diagnosed with stage III gastric cancer from November 2014 to December 2017 at Harbin Medical University Cancer Hospital. Detailed clinicopathological data were obtained from the patient’s medical records. The studies involving human participants were reviewed and approved by the Ethics Review Committee of Harbin Medical University Cancer Hospital (the ethics number: KY2021-09), and it adhered to the standards of the Declaration of Helsinki and its later amendments. The patients provided their written informed consent to participate in this study.

Participants were considered eligible if they were gastric cancer patients who: 1) were histologically diagnosed with stage III gastric cancer; 2) received primary tumor resection; 3) had no infection or inflammatory disorder; 4) had routine blood test performed at a week before treatment; and 5) had complete clinical recorded and follow-up data. The patient exclusion criteria were as follows: 1) malignant tumor at another site or multiple primary malignant tumors; 2) received anti-tumor therapy before surgery, including chemotherapy or targeted therapy; 3) liver and kidney dysfunction could not tolerate surgery; 4) chronic inflammatory disease or autoimmune disease; and 5) received the blood product transfusion within one month before surgery.

### Nutritional Risk Index (NRI)

The NRI, comprised three factors, was based on patients’ ideal body weight, present body weight (before surgery), and serum albumin levels in every patient. The NRI was calculated as follows: 1.519 × serum albumin level (g/l) + 41.7 × (present/ideal body weight). And the ideal weight (WLo) was calculated using the following formula: Height-100-[(Height-150)/2.5].

### Follow-Up

In the current study, disease-free survival (DFS)was defined as the time between the date of surgery and the time of progression with regard to recurrence or distant metastases, and all-cause death or the last follow-up. Overall survival (OS) was defined as the time between the date of surgery and all-cause death or the last follow-up. The last follow-up was assessed in December 2021. The survival data were through telephone interviews or extracted from telephone interviews.

### Statistical Analysis

The Chi-square test or Fisher’s exact test was used to analyze categorical variables, and t-tests were used to analyze continuous variables. Survival curves, including DFS and OS, were plotted by the Kaplan-Meier method, and the log-rank test was utilized to analyze the differences. The significant variables were identified from univariate and multivariate Cox proportional hazards regression model. The 95% confidence intervals (CIs) and hazard ratios (HRs) were performed to evaluate the association between patients’ NRI and prognosis. Nomogram for DFS and OS was established on the basis of the multivariate analyses. Statistical analysis data were statistically analyzed using SPSS 22.0 (SPSS Inc., Chicago, IL, USA) and R (version 3.6.0; Vienna, Austria. URL: http://www.R-project.org/). Each test was two-sided, and statistical differences were termed as *P* value < 0.05.

## Results

### Patient Characteristics

**Figure 1:**
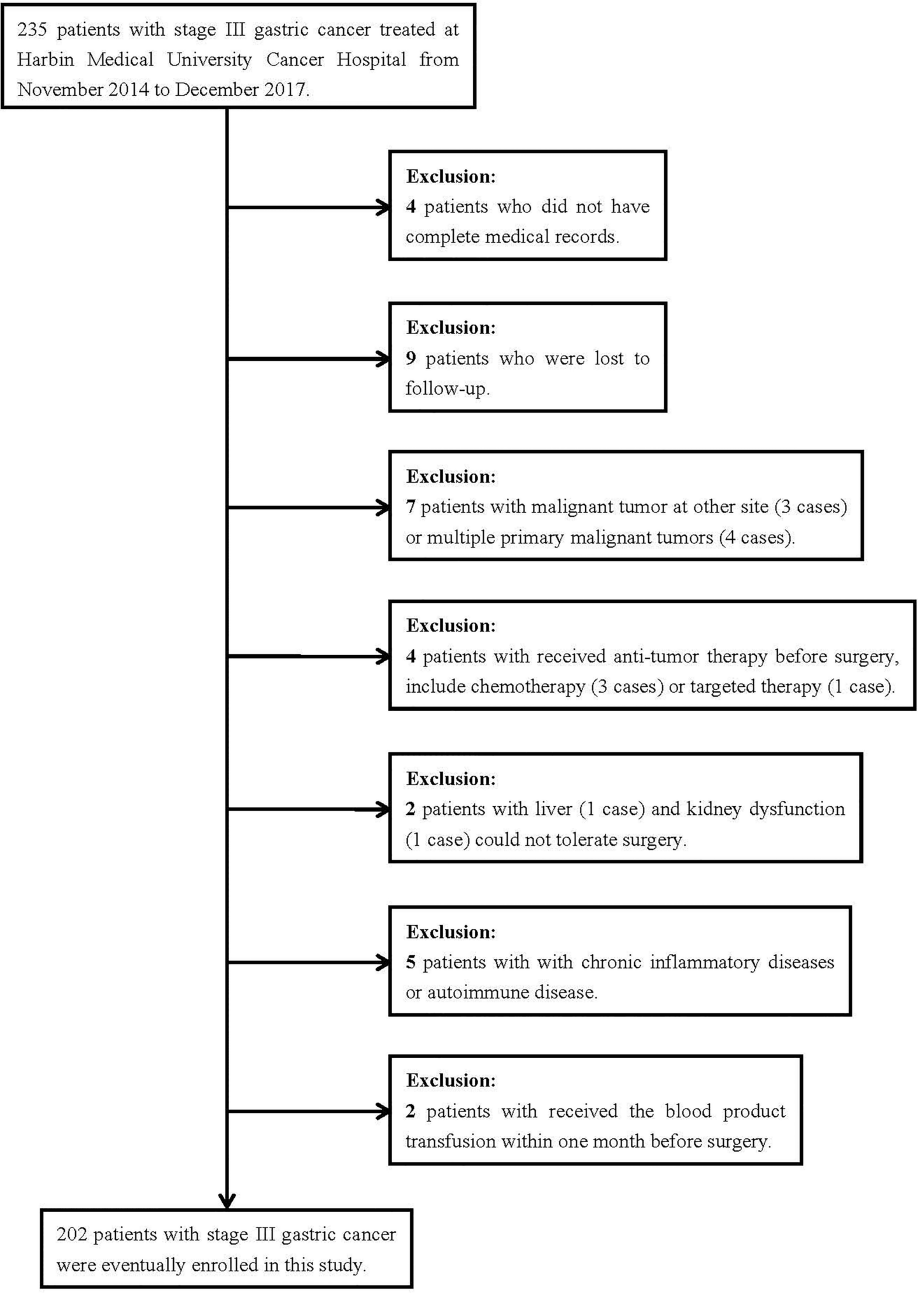
Flow diagram of the process of selection of the patients included in this study.

In total, 235 patients with stage III gastric cancer were treated at Harbin Medical University Cancer Hospital between November 2014 and December 2017. Through the inclusion and exclusion criteria, 202 patients were eventually enrolled, while the remaining 33 patients were excluded (**Figure 1**). There were 132 (65.3%) males and 70 (34.7%) females. The median age at the time of surgery of all cases was 61 (range from 28 to 83 years). The receiver operating characteristic curve (ROC) was used to determine the optimal cutoff value of NRI, and the value was 99. According to the optimal cutoff value of NRI, all patients were divided into two groups: low NRI group (NRI<99) and high NRI group (NRI≥99). The patient characteristics are shown in **Table 1**. With regard to patient characteristics, NRI showed a significant relationship with age, weightand, body mass index (BMI) (P<0.05).

**Table 1:** Association of NRI and patient characteristics.

Parameter n	Level	Overall	Low NRI	High NRI	p
		202	75	127	
Sex (%)	Male	132 (65.3)	45 (60.0)	87 (68.5)	0.283
	Female	70 (34.7)	30 (40.0)	40 (31.5)	
Age (median [IQR])		61.0 [52.0, 66.0]	62.0 [55.5, 69.0]	59.0 [50.5, 65.0]	0.025*
Age (%)	≤60	99 (49.0)	29 (38.7)	70 (55.1)	0.035*
	>60	103 (51.0)	46 (61.3)	57 (44.9)	
Personality type (%)	Extraversion	116 (57.4)	37 (49.3)	79 (62.2)	0.097
	Ambivert	26 (12.9)	9 (12.0)	17 (13.4)	
	Introversion	60 (29.7)	29 (38.7)	31 (24.4)	
WLo (%)	≤61	92 (45.5)	38 (50.7)	54 (42.5)	0.328
	>61	110 (54.5)	37 (49.3)	73 (57.5)	
Weight (%)	≤60	88 (43.6)	54 (72.0)	34 (26.8)	<0.001*
	>60	114 (56.4)	21 (28.0)	93 (73.2)	
Height (%)	≤168	92 (45.5)	38 (50.7)	54 (42.5)	0.328
	>168	110 (54.5)	37 (49.3)	73 (57.5)	
BMI (%)	≤22.04	98 (48.5)	60 (80.0)	38 (29.9)	<0.001*
	>22.04	104 (51.5)	15 (20.0)	89 (70.1)	
Drinking water (%)	Deep well water	100 (49.5)	37 (49.3)	63 (49.6)	1.000
	Surface water	102 (50.5)	38 (50.7)	64 (50.4)	
Speed of taking food (%)	Fast	84 (41.6)	29 (38.7)	55 (43.3)	0.402
	Middle	96 (47.5)	35 (46.7)	61 (48.0)	
	Slow	22 (10.9)	11 (14.7)	11 (8.7)	
Taste (%)	Salty	152 (75.2)	59 (78.7)	93 (73.2)	0.352
	Middle	31 (15.3)	8 (10.7)	23 (18.1)	
	Light	19 (9.4)	8 (10.7)	11 (8.7)	
ABO blood type (%)	A	74 (36.6)	31 (41.3)	43 (33.9)	0.494
	B	67 (33.2)	20 (26.7)	47 (37.0)	
	O	45 (22.3)	18 (24.0)	27 (21.3)	
	AB	16 (7.9)	6 (8.0)	10 (7.9)	
Radical resection (%)	R0	164 (81.2)	65 (86.7)	99 (78.0)	0.054
	R1	24 (11.9)	9 (12.0)	15 (11.8)	
	R2	14 (6.9)	1 (1.3)	13 (10.2)	
Type of surgery (%)	distal gastrectomy	161 (79.7)	64 (85.3)	97 (76.4)	0.053
	proximal gastrectomy	9 (4.5)	0 (0.0)	9 (7.1)	
	total gastrectomy	32 (15.8)	11 (14.7)	21 (16.5)	
Primary tumor site (%)	upper 1/3	18 (8.9)	3 (4.0)	15 (11.8)	0.091
	middle 1/3	21 (10.4)	6 (8.0)	15 (11.8)	
	low 1/3	136 (67.3)	52 (69.3)	84 (66.1)	
	whole	27 (13.4)	14 (18.7)	13 (10.2)	
Borrmann type (%)	Borrmann 0	2 (1.0)	0 (0.0)	2 (1.6)	0.624
	Borrmann I	7 (3.5)	4 (5.3)	3 (2.4)	
	Borrmann II	38 (18.8)	15 (20.0)	23 (18.1)	
	Borrmann III	134 (66.3)	48 (64.0)	86 (67.7)	
	Borrmann IV	16 (7.9)	7 (9.3)	9 (7.1)	
	Borrmann V	5 (2.5)	1 (1.3)	4 (3.1)	
Tumor size (%)	≤20mm	35 (17.3)	9 (12.0)	26 (20.5)	0.261
	>20 and <50mm	93 (46.0)	35 (46.7)	58 (45.7)	
	≥50mm	74 (36.6)	31 (41.3)	43 (33.9)	
Differentiation (%)	poorly differentiated	101 (50.0)	37 (49.3)	64 (50.4)	0.181
	moderately differentiated	99 (49.0)	36 (48.0)	63 (49.6)	
	well differentiated	2 (1.0)	2 (2.7)	0 (0.0)	
Pathology (%)	adenocarcinoma	71 (35.1)	21 (28.0)	50 (39.4)	0.159
	mucinous carcinoma	8 (4.0)	5 (6.7)	3 (2.4)	
	signet ring cell carcinoma	8 (4.0)	2 (2.7)	6 (4.7)	
	mixed carcinoma	113 (55.9)	47 (62.7)	66 (52.0)	
	others	2 (1.0)	0 (0.0)	2 (1.6)	
Lauren type (%)	Intestinal	90 (44.6)	27 (36.0)	63 (49.6)	0.136
	Diffuse	52 (25.7)	24 (32.0)	28 (22.0)	
	Mixed	60 (29.7)	24 (32.0)	36 (28.3)	
Postoperative chemotherapy (%)	No	95 (47.0)	33 (44.0)	62 (48.8)	0.605
	Yes	107 (53.0)	42 (56.0)	65 (51.2)	

NRI, nutritional risk index; WLo, ideal weight; BMI, body mass index. * With statistical differences (P < 0.05).

### Nutritional and Blood Parameters

All peripheral blood parameters and nutritional parameters were collected before surgery. Median values were used to group these indicators, including total protein (TP), albumin (ALB), Globulin (GLOB), prealbumin (PALB), glucose (Glu), cholesterol (CHOL), triglyceride (TRIG), white blood cell (W), neutrophils (N), lymphocyte (L), monocyte (M), hemoglobin (Hb), red blood cell (R), hematocrit (Hct), platelet (P), immunoglobulin A (IgA), immunoglobulin G (IgG), and immunoglobulin M (IgM). **Table 2** summarizes the relationship of NRI with nutritional and blood parameters. With regard to patient characteristics, peripheral blood parameters, and nutritional parameters, NRI showed a significant relationship with TP, ALB, A/G, PALB, Glu, W, N, L, Hb, R, and Hct, respectively (P<0.05). However, there were no significant differences in GLOB, CHOL, TRIG, M, P, IgA, IgG, and IgM between the two NRI groups (P>0.05).

**Table 2:** The relationships of NRI with nutritional and blood parameters.

Parameter	Level	Overall	Low NRI	High NRI	p
n		202	75	127	
TP (%)	≤67.00	100 (49.5)	56 (74.7)	44 (34.6)	<0.001*
	>67.00	102 (50.5)	19 (25.3)	83 (65.4)	
ALB (%)	≤40.00	91 (45.0)	60 (80.0)	31 (24.4)	<0.001*
	>40.00	111 (55.0)	15 (20.0)	96 (75.6)	
GLOB (%)	≤26.00	87 (43.1)	37 (49.3)	50 (39.4)	0.217
	>26.00	115 (56.9)	38 (50.7)	77 (60.6)	
A/G (%)	≤1.52	98 (48.5)	49 (65.3)	49 (38.6)	<0.001*
	>1.52	104 (51.5)	26 (34.7)	78 (61.4)	
PALB (%)	≤230.50	101 (50.0)	52 (69.3)	49 (38.6)	<0.001*
	>230.50	101 (50.0)	23 (30.7)	78 (61.4)	
Glu (%)	≤5.10	97 (48.0)	48 (64.0)	49 (38.6)	0.001*
	>5.10	105 (52.0)	27 (36.0)	78 (61.4)	
CHOL (%)	≤4.18	101 (50.0)	42 (56.0)	59 (46.5)	0.244
	>4.18	101 (50.0)	33 (44.0)	68 (53.5)	
TRIG (%)	≤1.10	99 (49.0)	44 (58.7)	55 (43.3)	0.050
	>1.10	103 (51.0)	31 (41.3)	72 (56.7)	
W (%)	≤6.46	100 (49.5)	50 (66.7)	50 (39.4)	<0.001*
	>6.46	102 (50.5)	25 (33.3)	77 (60.6)	
N (%)	≤3.71	101 (50.0)	46 (61.3)	55 (43.3)	0.020*
	>3.71	101 (50.0)	29 (38.7)	72 (56.7)	
L (%)	≤1.88	99 (49.0)	48 (64.0)	51 (40.2)	0.002*
	>1.88	103 (51.0)	27 (36.0)	76 (59.8)	
M (%)	≤0.46	100 (49.5)	43 (57.3)	57 (44.9)	0.118
	>0.46	102 (50.5)	32 (42.7)	70 (55.1)	
Hb (%)	≤134.20	100 (49.5)	51 (68.0)	49 (38.6)	<0.001*
	>134.20	102 (50.5)	24 (32.0)	78 (61.4)	
R (%)	≤4.32	100 (49.5)	48 (64.0)	52 (40.9)	0.003*
	>4.32	102 (50.5)	27 (36.0)	75 (59.1)	
Hct (%)	≤40.21	101 (50.0)	50 (66.7)	51 (40.2)	<0.001*
	>40.21	101 (50.0)	25 (33.3)	76 (59.8)	
P (%)	≤255.00	100 (49.5)	40 (53.3)	60 (47.2)	0.490
	>255.00	102 (50.5)	35 (46.7)	67 (52.8)	
IgA (%)	≤2.15	100 (49.5)	36 (48.0)	64 (50.4)	0.855
	>2.15	102 (50.5)	39 (52.0)	63 (49.6)	
IgG (%)	≤8.54	101 (50.0)	38 (50.7)	63 (49.6)	1.000
	>8.54	101 (50.0)	37 (49.3)	64 (50.4)	
IgM (%)	≤0.99	101 (50.0)	35 (46.7)	66 (52.0)	0.560
	>0.99	101 (50.0)	40 (53.3)	61 (48.0)	

TP, total protein; ALB, albumin; GLOB, Globulin; PALB, prealbumin; Glu, glucose; CHOL, cholesterol; TRIG, triglyceride; W, white blood cell; N, neutrophils; L, lymphocyte; M, monocyte; Hb, hemoglobin; R, red blood cell; Hct, hematocrit; P, platelet; IgA, immunoglobulin A; IgG, immunoglobulin G; IgM, immunoglobulin M. * With statistical differences (P < 0.05).

### Relationships of NRI With Pathological Characteristics

**Table 3** summarizes the relationship of NRI with pathological parameters. NRI showed a significant relationship with total lymph nodes (TLN) and human epidermal growth factor receptor 2 (HER2) (P<0.05). However, there were no significant differences in positive lymph nodes (PLN), Cytokeratin (CK), Vimentin, vascular endothelial growth factor (VEGF), Cluster of Differentiation 56 (CD56), Cluster of Differentiation 31 (CD31), Cluster of Differentiation 34 (CD34), D2-40, or S100 between the two NRI groups (P>0.05).

**Table 3:** The relationships of NRI with pathological parameters.

Parameter	Level	Overall	Low NRI	High NRI	p
n		202	75	127	
TLN (%)	≤28	98 (48.5)	27 (36.0)	71 (55.9)	0.010*
	>28	104 (51.5)	48 (64.0)	56 (44.1)	
PLN (%)	≤6	101 (50.0)	31 (41.3)	70 (55.1)	0.081
	>6	101 (50.0)	44 (58.7)	57 (44.9)	
HER2 (%)	Negative	189 (93.6)	66 (88.0)	123 (96.9)	0.029*
	Positive	13 (6.4)	9 (12.0)	4 (3.1)	
CK (%)	Negative	25 (12.4)	8 (10.7)	17 (13.4)	0.729
	Positive	177 (87.6)	67 (89.3)	110 (86.6)	
Vimentin (%)	Negative	189 (93.6)	71 (94.7)	118 (92.9)	0.846
	Positive	13 (6.4)	4 (5.3)	9 (7.1)	
VEGF (%)	Negative	171 (84.7)	62 (82.7)	109 (85.8)	0.689
	Positive	31 (15.3)	13 (17.3)	18 (14.2)	
CD56 (%)	Negative	201 (99.5)	75 (100.0)	126 (99.2)	1.000
	Positive	1 (0.5)	0 (0.0)	1 (0.8)	
CD31 (%)	Negative	173 (85.6)	61 (81.3)	112 (88.2)	0.256
	Positive	29 (14.4)	14 (18.7)	15 (11.8)	
CD34 (%)	Negative	110 (54.5)	42 (56.0)	68 (53.5)	0.847
	Positive	92 (45.5)	33 (44.0)	59 (46.5)	
D2-40 (%)	Negative	127 (62.9)	44 (58.7)	83 (65.4)	0.424
	Positive	75 (37.1)	31 (41.3)	44 (34.6)	
S100 (%)	Negative	32 (15.8)	8 (10.7)	24 (18.9)	0.177
	Positive	170 (84.2)	67 (89.3)	103 (81.1)	

TLN, total lymph nodes; PLN, positive lymph nodes; HER2, human epidermal growth factor receptor; CK, Cytokeratin; VEGF, vascular endothelial growth factor; CD56, Cluster of Differentiation 56; CD31, Cluster of Differentiation 31; CD34, Cluster of Differentiation 34. * With statistical differences (P < 0.05).

### Univariate and Multivariate Analyses on the Prognostic Predictors in Patients With Stage III Gastric Cancer

In univariate Cox regression analysis, NRI, A/G, PALB, FIB, Borrmann type, TLN, tumor size, S-100, and postoperative chemotherapy were related to the prognosis of gastric cancer patients for DFS, however, only NRI, FIB, Borrmann type, TLN, and tumor size were identified as the independent factor to predict the DFS upon multivariate analysis. In univariate Cox regression analysis, NRI, age, A/G, PALB, FIB, radical resection, type of surgery, Borrmann type, TLN, tumor size, CD56, S-100, and postoperative chemotherapy were associated with the prognosis of gastric cancer patients for OS, however, only NRI, type of surgery, TLN, tumor size, and CD56 were identified as the independent factors to predict the OS upon multivariate analysis. These results are shown in **Table 4**.

**Table 4:** Univariate and multivariate analyses on the prognostic predictors in patients with stage III gastric cancer.

Parameters	DFS					P Value	OS					P Value
		Univariate			Multivariate			Univariate			Multivariate	
	HR	95%CI	P Value	HR	95%CI		HR	95%CI	P Value	HR	95%CI	
NRI	0.591	0.389-0.899	0.014	0.637	0.385-0.955	0.038*	0.557	0.366-0.847	0.006	0.510	0.308-0.843	0.009*
Sex	1.155	0.750-1.779	0.512				1.147	0.745-1.767	0.533			
Age	1.510	0.985-2.314	0.059				1.678	1.095-2.573	0.018	1.279	0.799-2.047	0.305
Personality type	1.250	0.996-1.569	0.054				1.212	0.966-1.520	0.097			
WLo	0.806	0.531-1.224	0.312				0.832	0.548-1.264	0.389			
Weight	0.930	0.611-1.415	0.734				0.918	0.603-1.397	0.690			
Height	0.806	0.531-1.224	0.312				0.832	0.548-1.264	0.389			
BMI	1.131	0.744-1.719	0.565				1.141	0.750-1.735	0.538			
Drinking water	0.987	0.649-1.500	0.951				1.041	0.685-1.583	0.850			
Speed of taking food	1.074	0.787-1.467	0.652				1.042	0.765-1.420	0.793			
Taste	0.840	0.590-1.196	0.334				0.821	0.577-1.168	0.272			
TP	1.199	0.789-1.822	0.395				1.202	0.791-1.826	0.389			
ALB	0.966	0.635-1.470	0.872				0.969	0.637-1.475	0.884			
GLOB	1.498	0.972-2.308	0.067				1.462	0.949-2.252	0.085			
A/G	0.552	0.361-0.844	0.006	0.811	0.495-1.329	0.406	0.563	0.368-0.860	0.008	0.951	0.574-1.577	0.846
PALB	0.476	0.308-0.736	0.001	0.658	0.392-1.103	0.112	0.475	0.307-0.734	0.001	0.639	0.370-1.102	0.107
Glu	0.973	0.640-1.479	0.899				1.000	0.658-1.520	0.999			
CHOL	0.982	0.647-1.492	0.933				1.004	0.661-1.524	0.987			
TRIG	1.099	0.723-1.670	0.659				1.132	0.745-1.720	0.563			
ABO blood type	1.202	0.964-1.499	0.103				1.198	0.960-1.495	0.110			
W	0.973	0.640-1.478	0.898				0.947	0.623-1.438	0.798			
N	1.007	0.662-1.530	0.975				0.960	0.632-1.459	0.849			
L	0.765	0.503-1.164	0.211				0.800	0.526-1.218	0.298			
M	0.793	0.521-1.206	0.278				0.819	0.538-1.246	0.351			
Hb	1.321	0.868-2.010	0.194				1.269	0.834-1.930	0.266			
R	0.977	0.643-1.484	0.913				0.960	0.632-1.458	0.849			
Hct	1.012	0.666-1.538	0.954				0.958	0.631-1.456	0.841			
P	0.755	0.496-1.150	0.190				0.733	0.482-1.117	0.149			
INR	1.198	0.786-1.825	0.400				1.234	0.810-1.880	0.328			
FIB	1.842	1.201-2.824	0.005	1.588	1.011-2.493	0.045*	1.881	1.227-2.885	0.004	1.536	0.961-2.453	0.073
IgA	1.274	0.836-1.943	0.260				1.275	0.836-1.944	0.259			
IgG	1.033	0.679-1.571	0.881				1.035	0.681-1.575	0.871			
IgM	1.190	0.782-1.809	0.416				1.113	0.732-1.692	0.617			
Radical resection	1.590	1.156-2.187	0.004	1.358	0.935-1.972	0.108	1.678	1.215-2.317	0.002	1.469	0.991-2.178	0.055
Type of surgery	1.216	0.945-1.565	0.129				1.301	1.011-1.674	0.041	1.343	1.001-1.802	0.049
Primary tumor site	1.151	0.864-1.534	0.337				1.110	0.828-1.488	0.487			
Borrmann type	1.362	1.018-1.824	0.038	1.361	1.006-1.841	0.045*	1.340	1.007-1.784	0.045	1.294	0.942-1.778	0.111
TLN	0.616	0.404-0.940	0.024	0.588	0.368-0.939	0.026*	0.577	0.378-0.880	0.011	0.506	0.312-0.820	0.006*
PLN	1.206	0.794-1.832	0.380				1.137	0.749-1.728	0.546			
Tumor size	1.717	1.260-2.340	0.001	1.799	1.261-2.568	0.001*	1.669	1.221-2.282	0.001	1.921	1.311-2.816	0.001*
Differentiation	1.252	0.836-1.875	0.275				1.315	0.878-1.971	0.184			
Pathology	0.926	0.803-1.069	0.295				0.928	0.803-1.072	0.312			
Lauren type	0.865	0.677-1.107	0.250				0.857	0.668-1.100	0.225			
HER2	0.719	0.292-1.775	0.475				0.800	0.324-1.973	0.628			
CK	1.022	0.543-1.922	0.947				1.063	0.565-1.999	0.850			
Vimentin	1.811	0.874-3.752	0.110				1.576	0.762-3.263	0.220			
VEGF	1.265	0.736-2.176	0.395				1.360	0.791-2.339	0.266			
CD56	6.639	0.903-48.796	0.063				7.855	1.063-58.065	0.043	29.281	3.044-281.691	0.003*
CD31	0.828	0.450-1.523	0.543				0.939	0.509-1.729	0.839			
CD34	1.035	0.681-1.573	0.872				1.045	0.687-1.590	0.836			
D2-40	0.822	0.529-1.279	0.385				0.868	0.559-1.349	0.530			
S100	2.223	1.074-4.602	0.031	1.098	0.506-2.384	0.813	2.200	1.063-4.553	0.034	0.982	0.449-2.146	0.964
Postoperative chemotherapy	1.687	1.098-2.592	0.017	1.402	0.899-2.186	0.136	1.757	1.143-2.701	0.010	1.402	0.882-2.227	0.153

NRI, nutritional risk index; WLo, ideal weight; BMI, body mass index; TP, total protein; ALB, albumin; GLOB, Globulin; PALB, prealbumin; Glu, glucose; CHOL, cholesterol; TRIG, triglyceride; W, white blood cell; N, neutrophils; L, lymphocyte; M, monocyte; Hb, hemoglobin; R, red blood cell; Hct, hematocrit; P, platelet; IgA, immunoglobulin A; IgG, immunoglobulin G; IgM, immunoglobulin M; TLN, total lymph nodes; PLN, positive lymph nodes; HER2, human epidermal growth factor receptor; CK, Cytokeratin; VEGF, vascular endothelial growth factor; CD56, Cluster of Differentiation 56; CD31, Cluster of Differentiation 31; CD34, Cluster of Differentiation 34. * With statistical differences (P < 0.05).

### Survival Analysis and Prognostic Value of NRI

**Figure 2:**
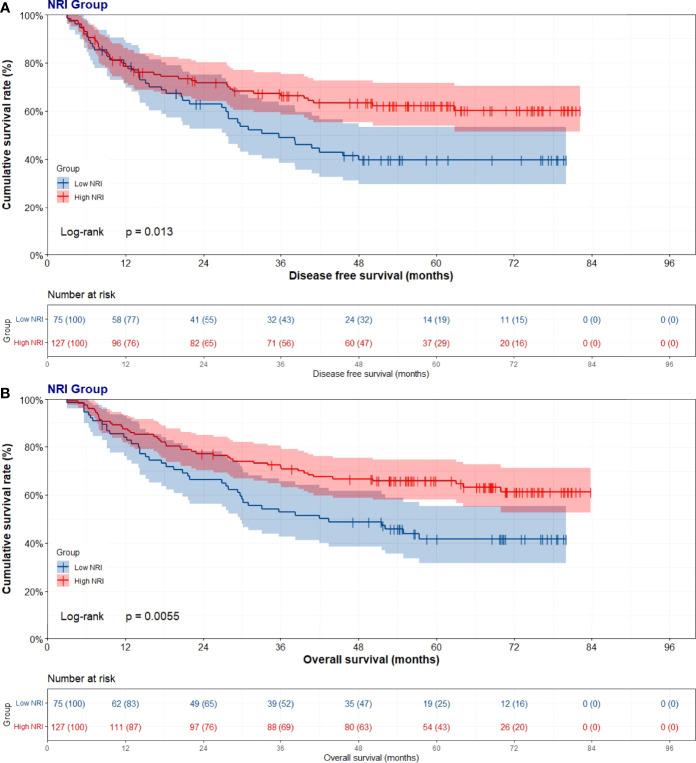
Disease free survival (DFS) and overall survival (OS) of patients with stage III gastric cancer. **(A)** Kaplan-Meier analysis of DFS for the NRI, **(B)** Kaplan-Meier analysis of OS.

Through the univariate and multivariate Cox regression analysis, the results indicated that high NRI was related to prolong DFS (P=0.014, HR: 0.591, 95% CI: 0.389-0.899 and P=0.038, HR: 0.637, 95% CI: 0.385-0.955) and OS (P=0.006, HR: 0.557, 95% CI: 0.366-0.847 and P=0.009, HR: 0.510, 95% CI: 0.308-0.843). The median DFS and OS in the low NRI group were 35.70 months and 43.40 months, respectively. The median DFS and OS in the high NRI group were not reached. Moreover, the median DFS and OS in the low NRI group were significantly shorter than that in the high NRI group (P=0.013 and P=0.0006), respectively (**Figure 2**).

**Figure 3:**
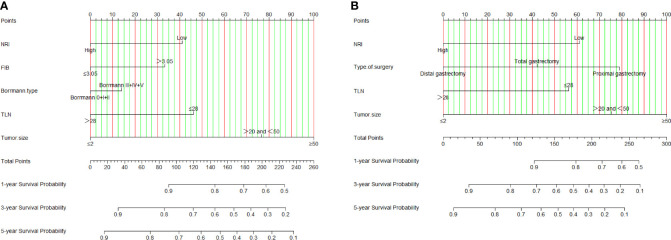
NRI-based nomogram for evaluating disease-free survival (DFS) and overall survival (OS). **(A)** NRI-based nomogram for evaluating DFS; **(B)** NRI-based nomogram for evaluating OS.

**Figure 4:**
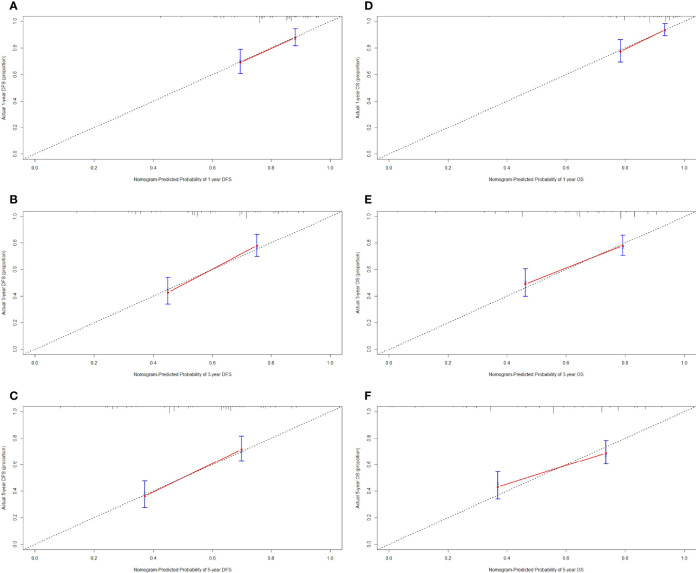
Calibration curve for predicting the 1-, 3-, 5-year disease-free survival (DFS) and overall survival (OS) rates. **(A)** 1-year DFS rate by calibration curve; **(B)** 3-year DFS rate by calibration curve; **(C)** 5-year DFS rate by calibration curve; **(D)** 1-year OS rate by calibration curve; **(E)** 3-year OS rate by calibration curve; **(F)** 5-year OS rate by calibration curve.

**Figure 5:**
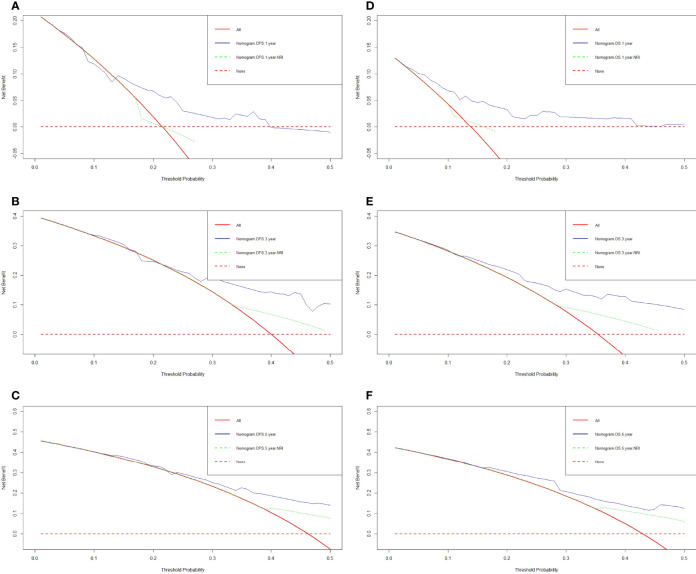
Decision curve analysis for the nomogram and only NRI. **(A)** 1-year DFS by decision curve analysis; **(B)** 3-year DFS by decision curve analysis; **(C)** 5-year DFS by decision curve analysis; **(D)** 1-year OS by decision curve analysis; **(E)** 3-year OS by decision curve analysis; **(F)** 5-year OS by decision curve analysis.

We constructed a nomogram for individualized assessment of DFS and OS after surgery. The nomogram for DFS had unique features, and integrated NRI, FIB, Borrmann type, TLN, and tumor size by the multivariate analysis. The nomogram for OS had unique features, and integrated NRI, type of surgery, TLN, and tumor size by the multivariate analysis. The nomogram of DFS and OS was generated as shown in **Figure 3**. Moreover, we used the calibration curve to evaluate the performance of the nomogram for predicted and the actual probability of survival time. The prediction line matches the reference line well for postoperative 1-, 3-, 5-year DFS and OS (**Figure 4**). Furthermore, the decision curve analysis (DCA) was performed to assess the clinical utility of the nomogram (the nomogram of DFS and OS by the multivariate analysis) and NRI by quantifying the net benefits at different threshold probabilities. Compared with only NRI, the nomogram model yielded the best net benefit across the range of threshold probability for 1-, 3-, 5-year DFS and OS, indicating its ability for clinical decision-making was better than only NRI (**Figure 5**).

## Discussion

Gastrectomy as a curative treatment of gastric cancer will lead to sustained weight loss, malnutrition, and then a decline in quality of life (21). Emerging evidence suggests that the prognosis of gastric cancer is not only associated with tumor indicators, but also related to systemic inflammation, patient’s condition, and nutritional status (22–24). Nowadays, due to the heterogeneity and comprehensiveness of gastric cancer, even if the same TNM is staged through the AJCC TNM staging system, the prognosis of patients may be different and vary greatly (25). As a result, it is necessary to develop an accurate prognostic risk stratification system to predict treatment outcomes.

Although some systemic inflammation or nutritional status indicators are used to assess the cancer prognosis, the single inflammation or nutrition-related marker may be misleading when the threshold is arbitrarily determined. Of late, a growing number of studies report that NRI, which is established based on serum albumin levels, present body weight, and ideal body weight, represents a novel nutrition-related prognostic scoring system. Researchers have also proven that NRI shows prognostic value for primary liver cancer, allogeneic hematopoietic cell transplantation (allo-HSCT), esophageal squamous cell carcinoma, and colorectal cancer (26–29). Besides, NRI is more accurate than other prognostic factors in predicting survival. For example, NRI was an independent prognostic factor for patients’ OS in a retrospective study centering on 143 patients with localized esophageal cancer (30). Another study indicated that NRI<100 in a baseline was significantly related to decreased OS in esophageal cancer patients of the SCOPE1 clinical trial (31). Furthermore, another study showed that GNRI was significantly associated with OS and cancer-specific survival (CSS) in elderly gastric cancer patients and was an independent predictor of OS; and is a simple, cost-effective, and promising nutritional index for predicting OS in elderly gastric cancer patients (32). A systematic review and meta-analysis showed that GNRI was a valuable predictor of complications and long-term outcomes in patients with gastrointestinal malignancy (33). However, there is little research on the role of NRI in predicting the prognosis of gastric cancer patients.

NRI is based on three factors, including serum albumin levels, present body weight, and ideal body weight. Nevertheless, little is known about the association between the NRI, treatment, and survival in patients with stage III gastric cancer. The current study was the first to evaluate the relationship between the NRI, clinicopathological factors, and prognosis. Our results proved that the high level of NRI was significantly related to age, weight, body mass index, TP, ALB, A/G, PALB, Glu, W, N, L, Hb, R, Hct, TLN, and HER2, respectively. Moreover, the NRI was a potential prognostic factor of DFS and OS by the univariate and multivariate Cox regression survival analyses. And the median DFS and OS in the high NRI group had longer survival than those in the high NRI group *via* the log-rank method. We also constructed a prognostic nomogram to predict the 1-, 3-, and 5-year survival probabilities, and the calibration curve shows that the prediction line matches the reference line well for 1-, 3-, and 5-year DFS and OS. Furthermore, the decision curve analysis also shows that the nomogram model yielded the best net benefit across the range of threshold probability for 1-, 3-, and 5-year DFS and OS compared to only NRI and indicated this model had better predicting ability for clinical decision-making.

There are several plausible mechanisms to explain the relationship between NRI and the prognosis of gastric cancer. The ALB is supposed to relate to the systemic inflammation affecting hepatocyte catabolism and anabolism (34). ALB also is one of the most common factors for determining the nutritional and immunological status (35). Patients with low ALB level go through poor hepatic functional reserve, which affects the tolerance to surgery and leads to worse survival time (36). BMI, defined as body mass in kilograms divided by the square of height in meters (kg/m^2^), is the most used anthropometric measure to approximate overall body fatness for the purposes of classifying and reporting overweight and obesity (37). Weight loss is common in advanced gastric cancer, and maintaining weight and adequate nutrition during systemic treatment (38). Moreover, the weight loss is usually caused by insufficient calorie intake as a result of tumor-related anorexia, malabsorption, hypermetabolism, and gastrointestinal obstruction (39).

Certain limitations should be noted in the current study. Firstly, this study was a single-center study with limited patients and also was a retrospective study. To further enrich the literature, multicenter studies from a large number of research populations should be enrolled. Secondly, as a result of the retrospective nature, selection bias was inevitable, although the enrolled patients were selected in line with the inclusion and exclusion criteria. Thirdly, NRI was a nonspecific tumor marker, and should further study the relationship between NRI, therapeutic effect, and prognosis in a prospective study.

## Conclusion

NRI is described as the potential prognostic factor for patients with stage III gastric cancer and is used to predict the survival and prognosis. The convenient, noninvasive, and reproducible factors are applied to guide treatment, evaluate efficacy, and estimate prognosis of gastric cancer.

## Data Availability Statement

The raw data supporting the conclusions of this article will be made available by the authors, without undue reservation.

## Ethics Statement

This study was reviewed and approved by the ethics committee of Harbin Medical University Cancer Hospital. The patients/participants provided their written informed consent to participate in this study.

## Author Contributions

HBS, HKS, and LY contributed to the study conception and design. YC, CY, and HX performed the collection of data. HG conducted the data interpretation. HBS prepared the manuscript. LL provided Funding acquisition and Project administration. All authors read and approved the final manuscript.

## Funding

This study was supported by the Funding of the Open Fund of Key Laboratory of Hepatosplenic Surgery, Ministry of Education, Harbin, China (No: GPKF202006 to LL) and the postdoctoral scientific research developmental fund from Heilongjiang province (No: LBH-Q19158 to LL).

## Conflict of Interest

The authors declare that the research was conducted in the absence of any commercial or financial relationships that could be construed as a potential conflict of interest.

## Publisher’s Note

All claims expressed in this article are solely those of the authors and do not necessarily represent those of their affiliated organizations, or those of the publisher, the editors and the reviewers. Any product that may be evaluated in this article, or claim that may be made by its manufacturer, is not guaranteed or endorsed by the publisher.
